# Mental health symptoms in German elite athletes: a network analysis

**DOI:** 10.3389/fpsyg.2023.1243804

**Published:** 2023-11-23

**Authors:** Sheila Geiger, Lisa Maria Jahre, Julia Aufderlandwehr, Julia Barbara Krakowczyk, Anna Julia Esser, Thomas Mühlbauer, Eva-Maria Skoda, Martin Teufel, Alexander Bäuerle

**Affiliations:** ^1^Clinic for Psychosomatic Medicine and Psychotherapy, LVR-University Hospital Essen, University of Duisburg-Essen, Essen, Germany; ^2^Center for Translational Neuro-and Behavioral Sciences (C-TNBS), University of Duisburg-Essen, Essen, Germany; ^3^Division of Movement and Training Sciences/Biomechanics of Sport, University of Duisburg-Essen, Essen, Germany

**Keywords:** mental health, sports, distress, depression, anxiety, somatic symptom disorder

## Abstract

**Introduction:**

Elite athletes are exposed to a variety of sport-specific stressors that may put them at particular risk for mental health symptoms and disorders. The aim of the present study was to assess data on mental health of elite athletes and investigate associations and interconnections among different variables using network analysis.

**Methods:**

A cross-sectional study was conducted from December 2021 to December 2022. The sample consisted of 275 German elite athletes (167 females) aged ≥18 years. Next to sociodemographic, medical and sport-related data, psychometric data such as psychological distress, symptoms of generalized anxiety, depression, and somatic symptom disorder have been gathered through questionnaires and analyzed by means of network analysis.

**Results:**

Over 95.0% of the athletes showed elevated distress and 28.6% reported symptoms of depression. Results of the network analysis show, among other findings, that symptoms of somatic symptom disorder were associated with severe injuries and substance use. Moreover, elite athletes who reported a better financial situation reported fewer symptoms of depression, generalized anxiety, and somatic symptom disorder. They also reported a lower incidence of mild to moderate injuries and severe injuries, fewer years spent in elite sports, less substance use, and fewer training sessions per week. Conversely, these athletes reported a higher level of distress. Furthermore, sex, financial situation and number of training units per week emerged as significant predictors for mental health symptoms.

**Discussion:**

Elite athletes showed increased numbers regarding mental health symptoms. Providing appropriate mental health interventions for elite athletes and further analysis of factors that influence the mental health of elite athletes and their interplay seem to be of central importance for the general well-being of elite athletes.

## 1 Introduction

Elite athletes often devote a significant amount of time and resources to their training and compete in events or competitions at national, international or professional levels (APA, 2023). In general, research supports the idea that physical activity has a positive impact on mental health (Daley, 2008; Hamer et al., 2009). Moreover, being involved in elite sports tends to have a beneficial impact on one’s lifespan (Lemez and Baker, 2015). However, on the other hand, some studies show that elite athletes can be particularly vulnerable to mental health symptoms and psychopathological disorders due to the unique stressors they face in competitive sports (Arnold and Fletcher, 2012; Gouttebarge et al., 2019). Intense physical activity at the level of elite sports can have a negative effect on mental wellbeing, potentially increasing anxiety, depression, overtraining, injury, and burnout (Peluso and Andrade, 2005). It is commonly believed that an excessive training load is a vital stimulus to improve athletic performance (Issurin, 2010). Nevertheless, an inadequate balance between training intensity and appropriate recovery periods may give rise to various impairments, such as overtraining syndrome or relative energy deficiency (Stellingwerff et al., 2021; Carrard et al., 2022). In addition to physical stress, elite athletes are also exposed to a range of risk factors such as performance pressure, injuries, competition pressure, risk of financial issues, lack of (social) support (Arnold and Fletcher, 2012), and involuntary retirement due to injuries (Gouttebarge et al., 2019). Female sex, young age, and injuries were found to be risk factors of anxiety symptoms (Rice et al., 2019). Moreover, psychosocial variables such as stress response can increase injury risk of elite athletes (Ivarsson et al., 2017).

Despite the risk factors and mental health symptoms, elite athletes are less likely to seek professional help for mental health symptoms compared to the general population (Watson, 2005). Reasons for this include fear of stigma and a potential impact on performance and career, a perception that seeking help is a sign of weakness as well as a lack of understanding of mental health (Gulliver et al., 2010, 2012). The high levels of training can lead to overexertion, which can manifest in a range of symptoms such as exhaustion, depression, anxiety disorders, hypertension, and a variety of somatic symptoms (Bompa et al., 2020). A systematic review and meta-analysis suggests that the prevalence of mental health symptoms and disorders may be slightly higher in current and former elite athletes (16.0–34.0%) compared to the general population (Gouttebarge et al., 2019). Moreover, one third of elite athletes reported symptoms of anxiety and/or depression, while 19.6% experienced distress (Gouttebarge et al., 2019). Thus, prevalences of mental health symptoms and disorders among elite athletes are comparable to, if not slightly higher than, those of the general population. However, their likelihood of seeking help is low, making them even more vulnerable to more severe and continued mental health symptoms. To date, we have not found any study that has investigated somatic symptom disorder in elite athletes. Given the physical and emotional demands of elite sports, which can both increase the risk of injury and cause mental health symptoms and the fact that competitive athletes need their bodies to perform the sports, an examination of the prevalence and severity of somatic symptom disorder in this population is warranted.

Mental health symptoms can lead to a variety of negative consequences such as decreased quality of life or increased risk for suicide (Evans et al., 2007; Moitra et al., 2021). Going further, there is evidence that mental health symptoms can negatively impact athletic performance (Beedie et al., 2000; Khan et al., 2017). Even though there has been a growing focus on identifying and addressing mental health symptoms among elite athletes in the past decade (Rice et al., 2016), the field of sports psychiatry and its associated research are still in their early stages. As a result, the mental health care currently provided to elite athletes may not consider sport-specific factors that could affect their risk for mental health symptoms or address unique diagnostic or treatment considerations for this group (Reardon and Factor, 2010; Bär and Markser, 2013). Additional information on these factors can be valuable for establishing preventive measures as well as managing acute phases.

To prevent mental health symptoms among athletes, it is important to identify and address potential risk and protective factors, as well as explore possible interconnections between relevant factors. Network analysis (NA) is an innovative way to depict and examine psychopathology and related constructs independent from categorial approaches (Levinson et al., 2018). NA is a method used to study complex systems or concepts by representing them as nodes in a network and examining the relationships between these nodes. The basis for this approach is network theory (Borsboom, 2017). Within the field of psychology, NA has been used to study a variety of topics, including the relationships between different symptoms of mental health disorders (Borsboom and Cramer, 2013). NA can be particularly useful in exploring relationships between complex concepts in an exploratory manner, without making any assumptions or hypotheses about the eventual network configuration (Borsboom and Cramer, 2013). Since psychological variables are assumed to influence each other directly and not to be caused by an unobserved latent entity (Borsboom and Cramer, 2013), NA represents the totality of interrelationships better than looking at partial aspects only. Moreover, NA enables us to examine numerous interrelationships simultaneously and to show how different aspects are interconnected in their entirety. We consider network analysis as a useful method to investigate these relationships in more detail. Levinson et al. suggest that psychological networks can further be used as important evidence base for more targeted treatment options, e.g., by focusing on central nodes in order to weaken the connections within the psychopathological network (Levinson et al., 2018). By uncovering key factors influencing the mental health of elite athletes and considering negative feedback processes between vulnerabilities, supportive and treatment offers can be adapted accordingly, to enhance their effectiveness.

The present study contains two objectives. The first one is to evaluate the prevalences and severity of generalized anxiety symptoms, depression symptoms, somatic symptom disorder symptoms and psychological distress in elite athletes. Similar to previous studies it is expected that there will be higher prevalences in mental health symptoms in elite athletes compared to the general population (Gouttebarge et al., 2019).

The second aim of this study is to identify different factors connected with mental health symptoms using NA and regression analyses. We focused on depressive symptoms, generalized anxiety, distress, financial situation, injuries and trainingload, as these are known risk factors and known mental health symptoms in the research (Gouttebarge et al., 2019; Rice et al., 2019; Stellingwerff et al., 2021; Carrard et al., 2022). Moreover, we included somatic symptom disorder and substance use as further variables because there has been little research to date on substance use among elite athletes and no research on somatic symptom disorder (Exner et al., 2021). Although NA is an exploratory method, it is anticipated that mental health symptoms will be linked to injuries (Gouttebarge et al., 2016; Kiliç et al., 2018). Additionally, there is an expectation that poorer financial circumstances are connected to mental health symptoms (Argabright et al., 2022; Guan et al., 2022).

By examining the potential factors linked to mental health symptoms in elite athletes, our findings could serve as a roadmap for future research that is relevant to clinical practice.

## 2 Methods

### 2.1 Study design and participants

This cross-sectional study was conducted in the form of a digital survey. It was performed within approval guidelines of the Ethics Committee of the Faculty of Medicine of the University of Duisburg-Essen (19-8947-BO). Prior to starting the survey, electronic informed consent was obtained from each participant. Participation was anonymous and voluntary. There was no form of reimbursement. The average time of completing the digital survey was 19:50 min. The standard deviation was 10:45 min and the range of time for completion amounted to 73:45 min. The Unipark software (Tivian XI GmbH) was used, and the survey was distributed from December 2021 to December 2022. The eligibility requirements included adult age (≥18 years), a good command of the German language, internet access, and being an elite athlete. An elite athlete was defined as a person who (1) aligns their life to sports, (2) strives for athletic excellence, and (3) participates in professional or Olympic competitions (Pfaff, 2004; Lämmle, 2011; Reardon et al., 2019). Sports federations (regional and national) and sports clubs (with elite athletes competing in regional and nationwide tournaments) of all sports were contacted via mail and social media. The criteria that had to be fulfilled in order to be considered an elite athlete and to be able to take part in the study were listed here. A total number of 382 participants answered the questionnaire. Out of these, 29 participants had to be excluded at the beginning of the survey because they were younger than 18 years. Another 78 participants were excluded from the study as they did not meet the criteria required for elite athlete status at the time of the survey. The final sample for data analysis consisted of *N* = 275 athletes.

### 2.2 Measures and psychometric instruments

The survey collected sociodemographic data, including age, sex, family status, housing situation, level of education, financial situation, and occupation. Medical data covered body height, body mass, medications, and injuries within the last 12 months (regardless of type, location, and whether or not it was a contact injury). Since the survey addressed elite athletes, it also covered different sport-related data such as type of sports, years in elite sports, number of training units per week, duration of training units, etc.

Five validated measures were used in the survey to assess aspects of mental health symptoms, namely generalized anxiety symptoms, depressive symptoms, somatic symptom disorder symptoms and psychological distress. First, the Generalized Anxiety Disorder Scale-7 (GAD-7) identifies the severity of generalized anxiety symptoms (Löwe et al., 2008). It consists of seven items assessing symptoms of generalized anxiety disorder over the past 2 weeks. Scores can range from 0 (not at all) to 3 (nearly every day) for each item. The total score ranges from 0 to 21 and can be categorized between minimal (0–4), mild (5–9), moderate (10–14), and severe (15–21) levels of anxiety. The GAD-7 is a reliable and valid tool to evaluate generalized anxiety symptoms (Löwe et al., 2008). Reaching a value of 0.85 for Cronbach’s alpha, this measure showed high internal consistency in our study. Second, the Patient Health Questionnaire-8 (PHQ-8) is a questionnaire to assess depression symptoms (Kroenke et al., 2008). It consists of eight items, each of which can score between 0 (not at all) and 3 (nearly every day), thus yielding a total score from 0 to 24. We used the recommended cut-off score of 10 points for major depression symptoms. The scale reached high internal consistency with Cronbach’s alpha of 0.85. Third, the Patient Health Questionnaire-15 (PHQ-15) and Somatic Symptom Disorder Scale (SSD-12) were used to assess somatic symptom disorder symptoms (Toussaint et al., 2020). The PHQ-15 evaluates the severity of somatic symptoms experienced during the past 4 weeks. Item scores range between 0 (not bothered at all) and 2 (bothered a lot) each, the sum score ranges between 0 and 30. It can be used to assess the A criteria of Somatic Symptom Disorder (SSD) defined by the Diagnostic and Statistical Manual of Mental Disorders (DSM-5). The recommend cut-off combined with SSD-12 is ≥9 (Toussaint et al., 2020). In our study, the PHQ-15 reached a value of 0.78 for Cronbach’s alpha, which is considered medium to high. Additionally, the SSD-12 was applied, which comprises 12 items to cover the B criteria of the DSM-5 definition of the SSD (excessive and disproportionate thoughts, feelings, and behaviors associated with somatic symptoms; Falkai et al., 2015). It has proven to be a reliable and valid tool to screen for the B criteria of SSD (Toussaint et al., 2017). Three subgroups containing four items each assess the aforementioned criteria. Item scores range from 0 to 4 with total sum scores ranging from 0 to 48. We used the optimal combined cut-off of ≥9 for the PHQ-15 and ≥23 for the SSD-12 (Toussaint et al., 2020). In our study, the SSD-12 reached a value of 0.90 for Cronbach’s alpha, which is considered excellent. Fourth, the Distress Thermometer (DT) was applied to evaluate psychological distress in the past week on a visual scale from 0 (no distress) to 10 (extreme distress). For the German version of the DT a cut-off of 4 points has been identified to be a sign of relevant distress (Mehnert et al., 2006).

### 2.3 Data analysis

Data analysis was conducted using SPSS Statistics 26 (IBM) and R 4.1.1 (R Core Team). Descriptive statistics were calculated for the participants’ sociodemographic data and scores on various psychometric instruments (GAD-7, PHQ-8, PHQ-15, SSD-12). Linear and multiple regression analyses were conducted to determine predictors of mental health variables. To provide evidence for the construct validity of the applied questionnaires, confirmatory factor analyses (CFA) were conducted. The maximum likelihood method was chosen as the estimation method. Model fit was assessed using a χ^2^ test and common approximate fit indices (RMSEA, SRMR, CFI, and TLI; Hu and Bentler, 1999). NA was performed using the packages qgraph, igraph, bootnet, and EGAnet (Csardi and Nepusz, 2006; Epskamp et al., 2012; Golino and Epskamp, 2017). Centrality indices were computed and assessment of the network’s stability and accuracy was conducted via bootnet. Missing data was addressed using listwise deletion, with the minimum sample size set to 250–350 participants to ensure sufficient power for the analysis of networks with 20 nodes or fewer (Constantin et al., 2021). The study estimated and visualized the network using a gaussian graphical model (Epskamp and Fried, 2018). Depressive symptoms, somatic symptom disorder, generalized anxiety, distress, mild to moderate injuries, severe injuries, years in elite sports, substance use, financial situation and training units per week were selected as nodes, resulting in a total of 11 nodes in the network. The dependencies among the variables were represented as edges in the network based on partial correlations (Epskamp and Fried, 2018). According to Epskamp and Fried (2018), gLASSO and EBIC (Chen and Chen, 2008; Friedman et al., 2008) methods were applied, with a tuning parameter of 0.5. The tuning parameter of 0.5 was chosen to create a parsimonious network with a higher specificity, as suggested by Epskamp and Fried (2018). The centrality indices were then calculated to determine the importance of each node in the network. These indices included degree centrality, strength, closeness, and betweenness (Hevey, 2018). Degree centrality is the sum of all edges of a node, strength is the sum of the edge weights of all edges of a node, closeness measures the average distance of a node to other nodes, and betweenness identifies the role of a node in connecting other nodes (Hevey, 2018). The centrality indices are intended to provide clues as to which constructs are particularly relevant in the context of – in this case - various mental health and sport-related variables (Epskamp and Fried, 2018). The stability and accuracy of the network were evaluated through different bootstrap procedures, including an edge weight variation analysis (Isvoranu et al., 2021) and a correlation stability analysis. It is recommended that, in order to interpret centrality with confidence, stability coefficients should exceed at least 0.25 and ideally surpass 0.50 (Epskamp et al., 2018). The interpretability of the edge weight, node strength, and centrality indices was also assessed.

## 3 Results

### 3.1 Sample characteristics

Table 1 shows the sample characteristics of the present study. Participants were between 18 and 63 years old (*M* = 23.67 years, *SD* = 6.29 years) with 76.5% being 18–25 years old. 60.7% of participants were female and 39.3% were male. The average body height for male athletes was 182.67 cm (*SD* = 7.44 cm), and their average body mass was 79.60 kg (*SD* = 11.36 kg). Female athletes had an average body height of 170.75 cm (*SD* = 7.23 cm) and an average body mass of 65.83 kg (*SD* = 8.56 kg). A total of 85 athletes took part in team sports only, while 143 were exclusively involved in individual sports, leaving 47 participants who were actively engaged in both. The number of athletes per type of sports is illustrated in Table 1. Most athletes practiced their sport for an average of 9.69 years (*SD* = 5.25) and, accordingly, began participating in elite sports at school age. Information regarding recent training habits reveals that the average length of each training session was 96.86 (*SD* = 32.96) minutes, and there were 8.20 (*SD* = 4.27) training units per week on average. The elite athletes exercised an average of 754.77 (*SD* = 367.42) minutes per week. No information was provided regarding whether the participants were affiliated with a German national team or if they were college or university students.

**Table 1:** Sample characteristics (*N* = 275).

Characteristic		*M* (*SD*)	*n*	%
Types of sports				
	Ball sports		90	32.8
	Combat sports		19	6.9
	Strengths sports		15	5.5
	Track and field		27	9.9
	Equestrian sports		17	6.2
	Gymnastics		11	4.0
	Dance sports		5	1.8
	Water sports		81	29.6
	Winter sports		2	0.7
	Trend sports		7	2.6
Days at home during last month				
		21.79 (7.42)		
Family status				
	Kids (underage)		15 (12)	5.5 (4.4)
	Single		187	68.0
	Partnership		63	22.9
	Married		21	7.6
	Divorced		2	0.7 0
	Widowed		0	0.0
	Other		2	0.7
Living situation				
	With parents		82	29.8
	alone		66	24.0
	Flat sharing		58	21.1
	With partner		49	17.8
	Alone with child(ren)		1	0.4
	With partner and child(ren)		12	4.4
	Sports center		4	1.5
	Other		3	1.1
Educational qualification				
	University education		63	22.9
	Higher education entrance qualification		159	57.8
	Vocational training		14	5.1
	Secondary Education		16	5.9
	Still in school education		15	5.5
	No school diploma		2	0.7
	Other		6	2.2
Employment status				
	Employed		48	22.9
	Self-employed		15	7.1
	Civil servant		11	5.2
	Other		26	12.4
Professional activity(ies) besides sports				
	Yes		210	76.4
	No		65	23.6
Earn a living through sports				
	Yes		53	19.3
	No		222	80.7
Mild injuries				
	Never		54	19.6
	1–2 times per year		112	40.7
	3–5 times per year		87	31.6
	6–20 times per year		18	6.5
	>20 times per years		4	1.5
Mild injuries				
	Never		137	49.8
	1–2 times per year		118	42.9
	3–5 times per year		20	7.3
Severe injuries				
	Never		219	79.6
	1–2 times per year		55	20.0
	>20 times per years		1	0.4
Operation necessary				
	Never		237	86.2
	1–2 times per year		37	13.5
	> 20 times per years		1	0.4

### 3.2 Confirmatory factor analyses

CFA could only confirm the three factorial structure of the SSD-12. Results of the CFA can be found in the Supplementary Table S6.

### 3.3 Prevalence of psychological distress, depression symptoms, generalized anxiety symptoms and somatic symptom disorder symptoms

The prevalence of psychological distress, depression symptoms, generalized anxiety symptoms, and somatic symptom disorder symptoms, stratified by sex is shown in Table 2. The GAD-7 (*M* = 5.0, *SD* = 3.8) measures revealed that 35.6% of participants had mild, 9.1% had moderate, and 2.9% had severe anxiety symptoms. Cut-off scores of PHQ-8 (*M* = 6.8, *SD* = 4.6) showed that 28.6% of the participants experienced symptoms of major depression. Using the combined cut-offs for the SSD-12 (≥23; *M* = 11.1, *SD* = 7.9) and the PHQ-15 (≥9; *M* = 7.0, *SD* = 4.3), 6.5% of participants exceeded the combined criterion, indicating somatic symptom disorder symptoms. The analyses of the DT scores (*M* = 7.4, *SD* = 2.0) resulted in 95.3% of participants reaching the cut-off value of 4, showing relevant distress. 95.3% reported any mental health symptoms and 4.7% reported none.

**Table 2:** Prevalence of generalized anxiety symptoms, depression symptoms, psychological distress and SSD12 stratified by sex.

		Sex	
		Female	Male
GAD-7			
<5	144 (52.4)	79 (47.3)	65 (60.2)
≥5	98 (35.6)	60 (35.9)	38 (35.2)
≥10	25 (9.1)	21 (12.6)	4 (3.7)
≥15	8 (2.9)	7 (4.2)	1 (0.9)
PHQ-8			
<10	196 (71.3)	117 (70.1)	79 (73.1)
≥10	79 (28.7)	50 (29.9)	29 (26.9)
PHQ-15/SSD-12			
<9; <23	257 (93.5)	152 (91.0)	105 (97.2)
≥9; ≥23	18 (6.5)	15 (9.0)	3 (2.8)
DT			
<4	13 (4.7)	10 (6.0)	3 (2.8)
≥4	262 (95.3)	157 (94.0)	105 (97.2)
Total	*N* = 275	*N* = 167	*N* = 108

Absolute values and percentage values in brackets. DT, distress; GAD7, Generalized anxiety symptoms, PHQ8, Depressive symptoms; PHQ15, Somatic Symptom Disorder symptoms; SSD12, Somatic Symptom Disorder symptoms.

### 3.4 Network estimation and visualization

**Figure 1:**
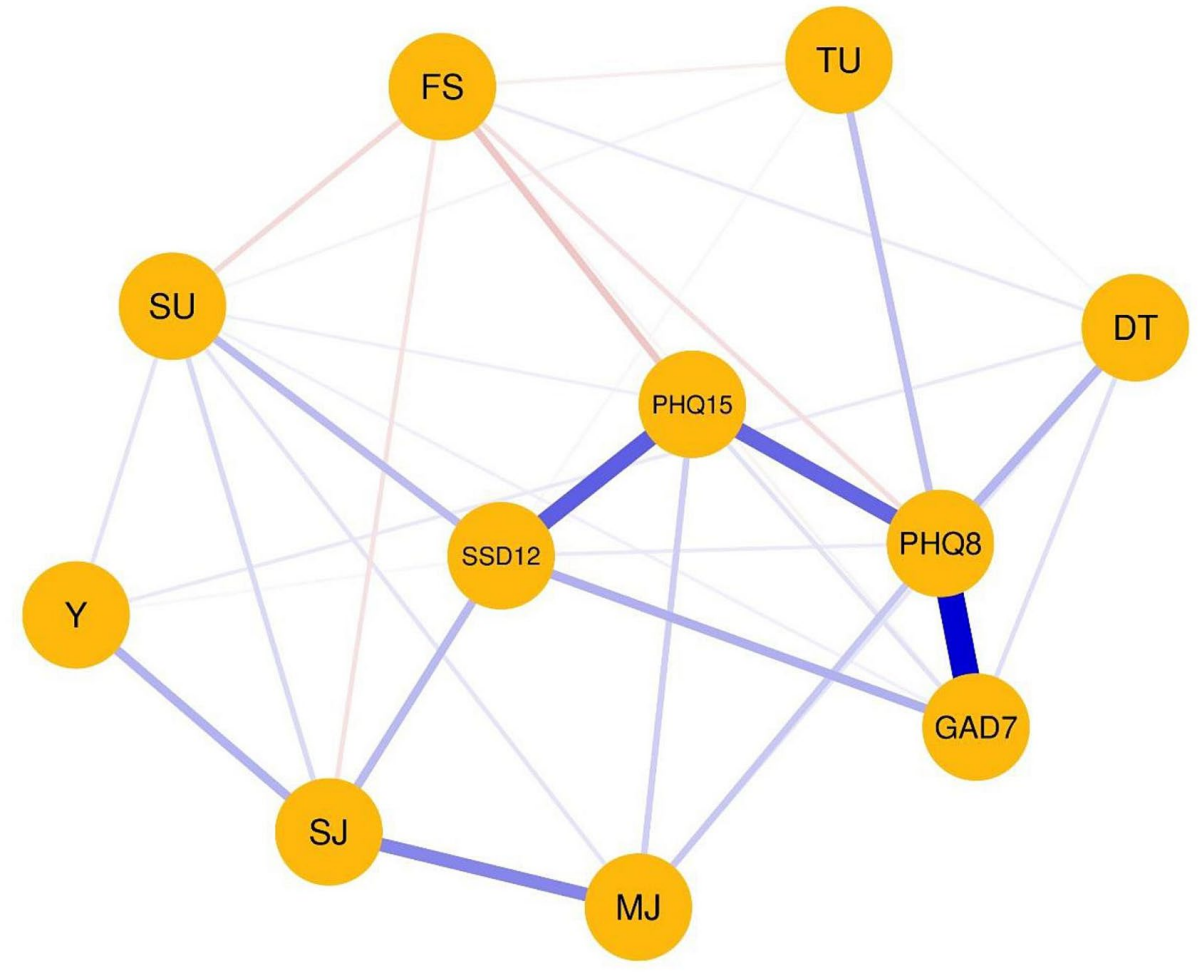
Visualized partial correlation network. The abbreviations within the network display the nodes. The thickness of the edges represents the edge connections between those nodes are referred to as edges. The thickness of the edges represents the edge weight, which is an indication of the strength of the edge. The thicker the edge, the higher the edge weight. Blue edges represent positive associations, whereas red edges represent negative associations. The meanings of the variables’ abbreviations can be seen on the right side of the network display. DT, distress; FS, financial situation; GAD7, Generalized anxiety symptoms; MJ, mild to moderate injuries; PHQ8, Depressive symptoms; PHQ15, Somatic Symptom Disorder symptoms; SSD12, Somatic Symptom Disorder symptoms; SJ, severe injuries to operation necessary; SU, substance use; TU, training units per week; Y, years in elite sports.

Figure 1 illustrates the Visualized Partial Correlation Network. After excluding 7 participants who had missing values on relevant variables, the network consisted of *N* = 268 participants. Across the network, 11 nodes were connected by 33 out of 55 (60.0%) possible edges. Among these, 27 displayed positive associations and 6 displayed negative ones. A graphical representation of the network with displayed edge weights can be found in the Supplementary Figure S1. The following strongest positive edges have been identified: Depression symptoms and generalized anxiety symptoms (0.55), somatic symptom disorder symptoms and severity of somatic symptoms (0.34), depression symptoms and severity of somatic symptoms (0.33), mild to moderate and severe injuries (0.26), somatic symptom disorder symptoms and generalized anxiety symptoms (0.17), years in elite sports and severe injuries (0.16), distress and depression symptoms (0.15), somatic symptom disorder symptoms and severe injuries (0.15), somatic symptom disorder symptoms and substance use (0.14) and training units per week and depression symptoms (0.13). The strongest negative edge weight was between financial situation and severity of somatic symptoms (−0.12). The edges associated with financial situation were generally all negative except for financial situation and distress (0.04).

### 3.5 Centrality indices

**Figure 2:**
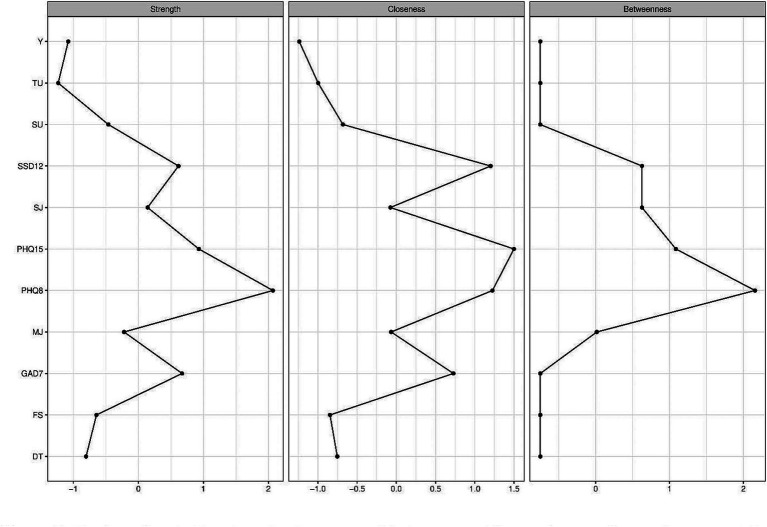
Display of centrality strength, closeness and betweenness. The numbers on the *y*-axis represent the allocated nodes numbered sequentially. The *x*-axis represents *z*-values associated with the centrality indices. The higher the *z*-value, the higher the centrality index of the respective variable. DT, distress; FS, financial situation; GAD7, Generalized anxiety symptoms; MJ, mild to moderate injuries; PHQ8, Depressive symptoms; PHQ15, Somatic Symptom Disorder symptoms; SSD12, Somatic Symptom Disorder symptoms; SJ, severe injuries to operation necessary; SU, substance use; TU, training units per week; Y, years in elite sports.

Figure 2 displays the centrality strength, closeness, and betweenness. The highest strength centrality was obtained for depression symptoms (1.23), severity of somatic symptoms (0.93), somatic symptom disorder symptoms (0.62), and generalized anxiety symptoms (0.67). The highest closeness index was found for severity of somatic symptoms (1.50), depression symptoms (1.23), somatic symptom disorder (1.20), and generalized anxiety symptoms (0.73). The highest betweenness index was detected for depression symptoms (2.16), severity of somatic symptoms (1.09), somatic symptom disorder (0.63), and severe injuries (0.63).

### 3.6 Network stability

To test whether the estimation of the partial correlation network was accurate and stable, bootstrap procedures were conducted for edge weight variation, the significance of edge weight and node strength differences, and the correlation stability of the centrality indices. Stability analysis revealed that the network model was stable. Edge weight stability of the network was good (0.67). The strength centrality coefficient and expected influence coefficient were excellent (0.75 for both). A graphical representation of the edge stabilities can be found in the Supplementary Figure S2.

### 3.7 Predictors of mental health symptoms

In the multiple regression analyses, it was shown that the sociodemographic variables and sport related variables significantly predicted depression symptoms (*R^2^* = 0.287; *F*_23,273_ = 4.384; *p* < 0.001). Of the sociodemographic variables female sex (*β* = 0.183; *p* = 0.002) and worse financial situation (*β* = −0.232; *p* = <0.001) significantly predicted depression symptoms. Of the sports related variables number of training units per week (*β* = 0.235; *p* = <0.001) and of the type of sports strength sports (*β* = 0.157; *p* = 0.047) significantly predicted depression symptoms.

Regarding the generalized anxiety disorder, sociodemographic variables and sports types represented significant predictors (*R^2^* = 0.246; *F*_23,273_ = 3.538; *p* < 0.001). Older age (*β* = 0.011; *p* = <0.001) and worse financial situation (*β* = −0.243; *p* = <0.001) as well as the living situations living alone (*β* = −0.274; *p* = 0.019) or living with parents (*β* = −0.290; *p* = 0.014) significantly predicted generalized anxiety symptoms. Also the type of sports, ball sports (*β* = 0.304; *p* = 0.017), strength sports (*β* = 0.229; *p* = 0.005) and track and field (*β* = 0.208; *p* = 0.024) represented significant predictors.

Only the sociodemographic factors female sex (PHQ-15: *β* = 0.248; *p* = <0.001; SSD-12: *β* = 0.229; *p* = 0.005) and worse financial situation (PHQ-15: *β* = −0.293; *p* = <0.001; SSD-12: *β* = 0.229; *p* = 0.005) and the sports related variable number of training units per week (PHQ-15: *β* = 0.160; *p* = 0.013; SSD-12: *β* = 0.229; *p* = 0.005) were significant predictors for somatic symptoms according to PHQ-15 (*R^2^* = 0.296; *F*_23,273_ = 4.568; *p* < 0.001) and SSD-12 (*R^2^* = 0.191; *F*_23,273_ = 2.568; *p* < 0.001).

There were no significant predictors in the multiple regression analysis of distress. All results of the regression analyses can be found in Supplementary Tables S1–S5.

## 4 Discussion

The present study examined the mental health and related factors of German elite athletes. Specifically, prevalences of generalized anxiety, depression, somatic symptom disorder, and psychological distress have been assessed among this population. An increased prevalence of generalized anxiety, depressive symptoms, and psychological problems in elite athletes has been shown. NA reveals strong associations between mental health symptoms. Somatic symptom disorder symptoms were associated with severe injuries and substance use. Furthermore, athletes who reported a more favorable financial situation reported fewer symptoms of depression, generalized anxiety, and somatic symptom disorder, as well as fewer mild to moderate injuries, severe injuries, years spent in elite sports, substance use, and training sessions per week. Conversely, these athletes reported higher levels of distress. Significant predictors for mental health symptoms were sex, financial situation and training units per week.

### 4.1 Prevalence of mental health symptoms

In the study sample, 35.6% showed mild symptoms of generalized anxiety disorder. Moderate levels were observed in 9.1% of the participants, three times higher than the average (3.0%) in the German population (Löwe et al., 2008). Severe symptoms were found in 2.9% of the participants, surpassing the rates of 1.0 and 1.2% reported in a normative sample and a population-based study, respectively (Löwe et al., 2008; Hinz et al., 2017). These results are consistent with research showing a 33% prevalence of depression/anxiety (undifferentiated) in elite athletes (Gouttebarge et al., 2019). Moreover, this study found that 28.6% of the participants experience symptoms of major depression, which aligns with current research (Gouttebarge et al., 2019). In a large random comparison sample (*N* = 198,678), 8.6% of the participants experienced depressive symptoms (Kroenke et al., 2008). Thus, elite athletes report higher prevalences of depressive symptoms than the general population in Germany. Furthermore, 6.5% of participants showed symptoms of somatic symptom disorder, which is in line with the estimated prevalence of 5.0–7.0% in the general population in Germany (Toussaint et al., 2020). This suggests that elite athletes are not necessarily more likely to experience somatic symptom disorder than the general population. One potential reason for this is that in somatic symptom disorder, one source of stress may be the presence of physical symptoms without any clear medical explanation (APA, 2000). In contrast, athletes often have a clear understanding of the physical symptoms they experience and may not face an additional psychological burden as a result. The prevalence of psychological distress was found to be considerably higher (95.3% versus 39.0%), compared to samples from the German population and to those found in the meta-analysis of Gouttebarge and colleagues, in which on average 19.6% of elite athletes reported distress (Gouttebarge et al., 2019; Hinz et al., 2019). One explanation could be that the elite athletes understood distress as purely physical distress and indicated a high level of distress due to the physical stress of the training. Another explanation could be that elite athletes tend to experience a lot of arousal in order to perform best at competition (Raglin, 1992). One must also consider that high between-study heterogeneity was observed in the study of Gouttebarge and colleagues, likely due to measurement-related concerns and differences in the cohorts (Gouttebarge et al., 2019).

### 4.2 Mental health symptoms and injuries in elite athletes

Results of the NA reveal close connections between mental health symptoms and injuries due to sports. Specifically, included were symptoms of generalized anxiety, depression, and somatic symptom disorder, as well as injuries. The results are consistent with recent research finding a higher overall risk for mental health symptoms and disorders in elite athletes with severe musculoskeletal injuries and multiple surgeries (Gouttebarge et al., 2016; Kiliç et al., 2018). This suggests that injuries and mental health symptoms are interconnected. Elite athletes are exposed to high levels of physical demands throughout their careers and this cumulative physical load, when combined with inadequate recovery, puts them at risk of developing musculoskeletal injuries (Kenttä and Hassmen, 1998; Chen et al., 2005; Cunniffe et al., 2009). In line with this, NA shows that severe injuries were associated with years in elite sports. Thus, addressing mental health in athletes requires a multifaceted approach that includes both providing support and resources for athletes who have suffered injuries (Putukian, 2016; Rice et al., 2018) and even more important proactively addressing the root causes of mental health symptoms.

### 4.3 Interconnections between mental health symptoms and financial situation

Furthermore, NA demonstrates a strong correlation between depressive symptoms and generalized anxiety, indicating that these conditions are closely intertwined and are likely to occur together. This finding is consistent with previous research that highlights a high comorbidity of depression and anxiety at both the disorder and symptom levels both in the general population and in elite athletes (Jacobson and Newman, 2017; Åkesdotter et al., 2022). This corresponds with the centrality analysis results. In particular, mental health variables show high centrality in the network analysis. The dominance of mental health variables in centrality was expected due to their frequency in the network analysis. Nevertheless, the result confirms our expectations and means that no other variable has a higher degree of centrality. Furthermore, a more favorable financial situation was associated with fewer symptoms of depression, generalized anxiety, and somatic symptom disorder. Less financial burden was further related to fewer mild to moderate injuries, severe injuries, fewer years of involvement in elite sports, and lower levels of substance use and training sessions per week. Conversely, athletes with less financial burden reported higher levels of distress, which is an unexpected finding. Financial stress has been proposed as an economic determinant of depression (Guan et al., 2022) and higher financial worries were significantly associated with higher psychological distress (Ryu and Fan, 2023). In the present study, the results were the opposite. The less financial stress, the more distress was reported by the elite athletes. One explanation could be that athletes who have a better financial situation tend to work more and thus experience more distress.

### 4.4 Predictors of mental health symptoms

In the multiple regression analyses sex, financial situation, training units and strength sports were significant predictors of depression symptoms. Female sex, worse financial situation, more training units and engaging in strength sports were associated with more depression symptoms. Moreover, age, financial situation, living alone, living with parents, ball sports, strength sports and track and field were found to significantly predict generalized anxiety symptoms. Financial situation, living alone and living with parents were associated with less generalized anxiety symptoms. Engaging in ball sports, strength sports and track and field was associated with more generalized anxiety symptoms. Fittingly, in a recent study, female sex was found to be a significant predictor of depressive symptoms, while age was not found to have significant predictive value (Wilson et al., 2022). In line with this, a meta-analysis showed that male elite athletes exhibited a 52% lower likelihood of reporting mild or severe depressive symptoms compared to their female counterparts who are also elite athletes (Gorczynski et al., 2017). This association also exists in the general population (Parker and Brotchie, 2010), highlighting the established sex disparity in relation to depressive symptoms. Moreover financial concern was found to be a significant predictor of both depression symptoms and anxiety (Pensgaard et al., 2021). These findings are also consistent with the outcomes from the network analysis. Additionally, financial situation appears to be a predictor of somatic symptom disorder. Worse financial situation was associated with more symptoms somatic symptom disorder. The financial situation, respectively the financial burden, seems to be an important factor regarding the mental health of elite athletes. Unlike our findings, a recent study did not find age and sports type to be significant predictors of generalized anxiety (Li et al., 2021). However, sex was not a significant predictor of generalized anxiety in this study, consistent with our results (Li et al., 2021). Interestingly, both living alone and living with parents were shown to be significant predictors of generalized anxiety. This finding suggests further investigation into the potential factors or dynamics associated with these life situations that might contribute to the experience of generalized anxiety. Furthermore, sex, financial situation, and training units per week emerged to be significant predictors for somatic symptom disorder. Female sex, worse financial situation and more training units were associated with more symptoms of somatic symptom disorder. Thus, training units per week was a significant predictor for both symptoms of depression and somatic symptom disorder. However, although current research shows that physical activity has positive effects on psychological well-being (Daley, 2008; Hamer et al., 2009), one explanation for this result could be overtraining syndrome. Overtraining syndrome refers to a condition characterized by a long-term decline in performance that occurs after a sustained imbalance between exercise-related and non-exercise-related stress and recovery (Meeusen et al., 2013). Insufficient focus on maintaining a proper balance between training intensity and recovery can result in enduring fatigue and unusual training responses (maladaptation) (Fry et al., 1991; Kenttä and Hassmén, 1998; Meeusen et al., 2013). In summary, the complex interplay of various factors such as sex, financial situation, and training units per week, as highlighted in the regression analyses, underscores the importance of considering these elements when understanding the mental health of elite athletes.

### 4.5 Limitations and strengths

The study design was cross-sectional, which precludes causal inference from the data. Additionally, all data was obtained via self-reporting, which makes objective verification of symptoms impossible. The data was collected through an online survey that was distributed via social media, sports clubs, and sports associations and thus selection bias should be taken into consideration when interpreting the findings. Moreover, retest-reliability assessment was not possible; future research should address this limitation to strengthen findings with elite athletes. Another important limitation is the construct validity assessment of the measurement instruments, especially for elite athletes. While we conducted confirmatory factor analyses and reliability assessments, the use of elite athlete-specific validated instruments is a consideration. The contrast between assumed and athlete-observed structures requires careful interpretation. Future studies could develop and validate measurement tools for elite athletes’ unique mental health dynamics. Similarly, the lack of cut-off values specifically validated for elite athletes in the questionnaires used is an additional limitation that needs to be addressed. Again, future research is needed to establish appropriate cut-off values for elite athletes. Consequently, the results can only be interpreted in the context of studies that examined different populations. Furthermore, the present study had a higher proportion of female participants and athletes with higher education levels, which should also be considered in terms of generalizability, as research shows that female elite athletes report performance anxiety, poor concentration, and somatic anxiety to a higher degree than males (Abrahamsen et al., 2008). Although comparative studies utilized highly validated and representative reference values, the differing age and sex distributions in the reference samples represent a limitation. Missing data on elite athletes’ specific sports, regional backgrounds (i.e., East or West Germany) and performance/competition level (e.g., highest level throughout career and current level) leads to uncertainties regarding the scope of the study. Future research should incorporate these variables for a more comprehensive analysis. Furthermore, the scheme used for classifying types of sports in Table 1, while practical for data collection, exhibited limitations due to inconsistent criteria and overlapping categories. Adopting the analytically clear categorization proposed by Emrich et al. (2001) in future studies could offer more precise insights into the relationships between different sport types and mental health symptoms. The timespan in which our data was collected (i.e., December 2021 to December 2022) may also be of consequence, as the fading COVID-19 pandemic may have affected prevalence of mental health symptoms (Dragioti et al., 2022; Robinson et al., 2022). To the best of our knowledge, this study is the first to offer a NA of mental health symptoms in elite athletes. Furthermore, we were able to survey a relatively large sample size.

## 5 Conclusion

In conclusion, the results of this study suggest that elite athletes have higher prevalences of mental health symptoms compared to the general population. The findings suggest that elite athletes may face unique challenges related to their sports, such as injuries and financial stressors, which may contribute to their risk for mental health symptoms. The high rates of psychological distress, depression and anxiety highlight the need for increased awareness and the importance of providing access to mental health resources for elite athletes, especially when they are at risk for or recovering from injury. To effectively address mental health symptoms in elite athletes, it is important to focus not only on treating symptoms, but also to explore the underlying causes that contribute to these mental health symptoms. Sex, financial situation, and number of training sessions seem to be essential factors in this context. Since many symptoms are closely related to each other, it is important to respond as soon as some of them occur. Early intervention could include screening programs, education and training for athletes and coaches, access to mental health professionals and to eHealth interventions. Further research is needed to better understand the factors contributing to the high levels of mental health symptoms seen in elite athletes.

## Data availability statement

The raw data supporting the conclusions of this article will be made available upon reasonable request to the corresponding author.

## Ethics statement

The studies involving humans were approved by Ethics Committee of the Faculty of Medicine of the University of Duisburg-Essen. The studies were conducted in accordance with the local legislation and institutional requirements. The participants provided their electronic informed consent to participate in this study.

## Author contributions

AB, MT, E-MS, and SG initiated and conceptualized the study. JA and TM were co-responsible for the recruitment of the participants. SG, LJ, and JK performed the statistical analyses and interpretation of the data. SG wrote the first draft of the manuscript. All authors contributed to the further writing of the manuscript and approved the final version.
