# Supplementary material for: Mental health symptoms in German elite athletes: a network analysis
Source: Front Psychol. 2023 Nov 23;14:1243804. doi: 10.3389/fpsyg.2023.1243804 (PMC10706480; doi:10.3389/fpsyg.2023.1243804)
Supplement: Supplementary file 1 [file Data_Sheet_1.docx]

Supplementary Material

**Mental Health Symptoms in German Elite Athletes: A Network Analysis**

Sheila Geiger^1,2*^, Lisa Maria Jahre^1,2^, Julia Aufderlandwehr^1^, Julia Barbara Krakowczyk^1,2^, Anna Julia Esser^1,2^, Thomas Muehlbauer^3^, Eva-Maria Skoda^1,2^, Martin Teufel^1,2^, Alexander Baeuerle^1,2^

*** Correspondence:** Sheila Geiger: [sheila.geiger@uni-due.de](mailto:sheila.geiger@uni-due.de)

# Supplementary Figures and Tables

## Supplementary Figures

**
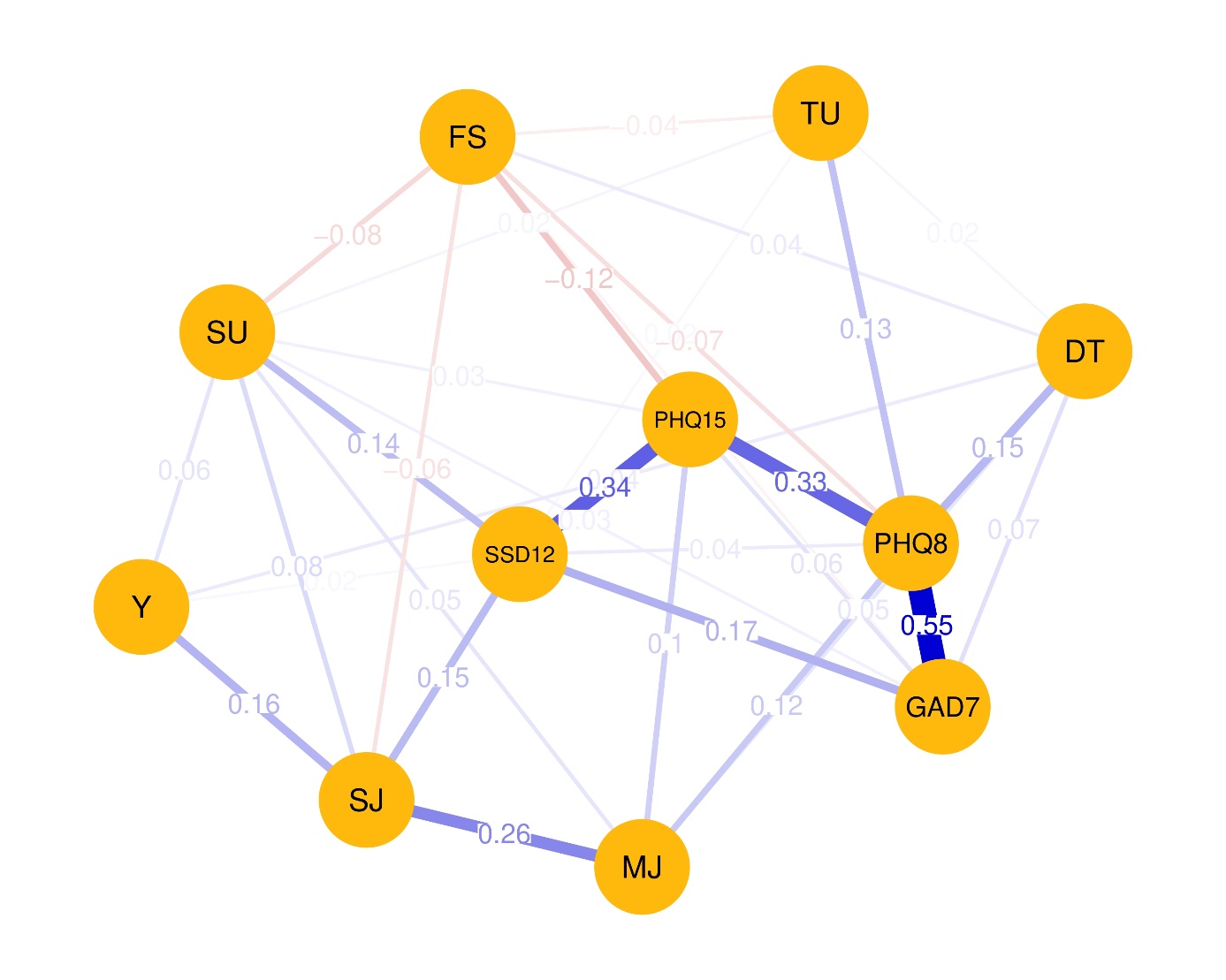
**

**Supplementary Figure 1.** Visualized Partial Correlation Network. The abbreviations within the network display the nodes. The thickness of the edges represents the edge connections between those nodes are referred to as edges. The thickness of the edges represents the edge weight, which is an indication of the strength of the edge. The thicker the edge, the higher the edge weight. Blue edges represent positive associations, whereas red edges represent negative associations. The meanings of the variables’ abbreviations can be seen on the right side of the network display. FS: financial situation, TU: training units per week, SU: substance use, Y: years in elite sports, DT: distress, PHQ8: Depressive symptoms, GAD7: Generalized Anxiety symptoms, SSD12: Somatic Symptom Disorder symptoms, PHQ15: Somatic Symptom Disorder symptoms, MJ: mild to moderate injuries, SJ: severe injuries to operation necessary.


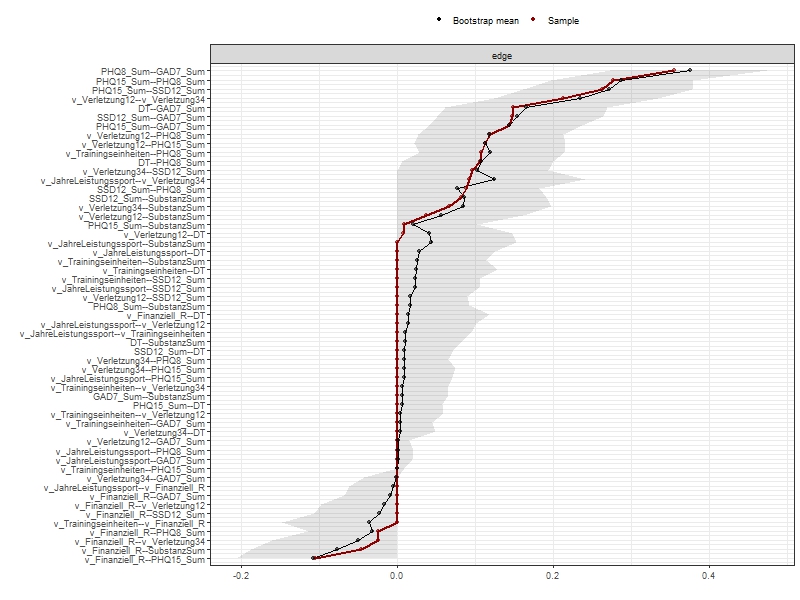


**Supplementary Figure 2**. Bootstrapped edge-weights for the network are displayed as black line. Point estimates of the edge weights are visualized by the red line. 95% confidence intervals are represented in grey.

## Supplementary Tables

**Supplementary Table 1.** Regression models of PHQ-8

| **Predictor** |  |  |  |  |  |  |
| --- | --- | --- | --- | --- | --- | --- |
|  | ***β*** | **B** | **T** | ***R*²** | **Δ *R*²** | ***P* value** |
|  | | |  |  |  |  |
| **Sociodemographic predictors** | | |  | .287 | .222 |  |
| Sex | .183 | 1.736 | 3.197 |  |  | .002 |
| Age | -.133 | -.098 | -1.679 |  |  | .094 |
| Earn a living | .079 | .929 | 1.353 |  |  | .177 |
| Financial situation | -.232 | -.516 | -3.961 |  |  | <.001 |
| Professional activity(ies)  besides sports | -.036 | -.395 | -.634 |  |  | .527 |
| Family status |  |  |  |  |  |  |
| Single | -.173 | -1.716 | -.891 |  |  | .374 |
| Married | -.113 | -1.978 | -.815 |  |  | .416 |
| Partnership | .189 | -2.088 | -1.034 |  |  | .302 |
| Living situation |  |  |  |  |  |  |
| Alone | -.120 | -1.301 | -1.064 |  |  | .288 |
| With partner | -.139 | -1.691 | -1.243 |  |  | .215 |
| With partner and child(ren) | -.049 | -1.101 | -.511 |  |  | .610 |
| With parents | -.175 | -1.816 | -1.526 |  |  | .128 |
| Flat sharing | .009 | .102 | .084 |  |  | .933 |
| **Sports related variables** | | |  |  |  |  |
| Years in elite sport | .077 | .068 | 1.145 |  |  | .253 |
| Number of training units  per week | .235 | .256 | 3.646 |  |  | <.001 |
| Duration of training units | .000 | -2.324 | -.003 |  |  | .988 |
| Types of sports |  |  |  |  |  |  |
| Ball sports | .220 | 2.174 | 1.781 |  |  | .076 |
| Combat sports | .096 | 1.746 | 1.178 |  |  | .240 |
| Strength sports | .157 | 3.210 | 1.997 |  |  | .047 |
| Track and field | .139 | 2.156 | 1.559 |  |  | .120 |
| Equestrian sports | -.121 | -2.320 | -1.495 |  |  | .136 |
| Gymnastics | -.013 | -.301 | -.177 |  |  | .859 |
| Water sports | .009 | .093 | .073 |  |  | .942 |

Note. *N* = 275. *β*: standardized coefficient beta; B: unstandardized coefficient beta; *R*²: determination coefficient; Δ *R*²: Changes in *R*²; PHQ-8: Depressive symptoms.

**Supplementary Table 2.** Regression models of GAD-7

| **Predictor** |  |  |  |  |  |  |
| --- | --- | --- | --- | --- | --- | --- |
|  | ***β*** | **B** | **T** | ***R*²** | **Δ *R*²** | ***P* value** |
|  | | |  |  |  |  |
| **Sociodemographic predictors** | | |  | .246 | .176 |  |
| Sex | .242 | 1.895 | 4.116 |  |  | .892 |
| Age | .011 | -.007 | .136 |  |  | <.001 |
| Earn a living | .063 | .610 | 1.049 |  |  | .295 |
| Financial situation | -.243 | -.232 | -4.043 |  |  | <.001 |
| Professional activity(ies)  besides sports | -.042 | -.036 | -.722 |  |  | .471 |
| Family status |  |  |  |  |  |  |
| Single | .012 | .097 | .059 |  |  | .953 |
| Married | -.129 | -1.848 | -.898 |  |  | .370 |
| Partnership | -.067 | -.607 | -.354 |  |  | .723 |
| Living situation |  |  |  |  |  |  |
| Alone | -.274 | -2.445 | -2.360 |  |  | .019 |
| With partner | -.220 | -2.211 | -1.918 |  |  | .056 |
| With partner and child(ren) | -.059 | -1.096 | -.601 |  |  | .548 |
| With parents | -.290 | -2.484 | -2.463 |  |  | .014 |
| Flat sharing | -.118 | -1.108 | -1.079 |  |  | .282 |
| **Sports related variables** | | |  |  |  |  |
| Years in elite sport | .056 | .077 | .817 |  |  | .415 |
| Number of training units  per week | .096 | .235 | 1.441 |  |  | .151 |
| Duration of training units | -.009 | .000 | -.154 |  |  | .878 |
| Type of sports |  |  |  |  |  |  |
| Ball sports | .304 | 2.477 | 2.394 |  |  | .017 |
| Combat sports | .075 | 1.135 | .904 |  |  | .367 |
| Strength sports | .229 | 3.842 | 2.820 |  |  | .005 |
| Track and field | .208 | 2.660 | 2.269 |  |  | .024 |
| Equestrian sports | -.021 | -.337 | -.256 |  |  | .798 |
| Gymnastics | .037 | .727 | -.506 |  |  | .613 |
| Water sports | .112 | .941 | .876 |  |  | .382 |

Note. *N* = 275. *β*: standardized coefficient beta; B: unstandardized coefficient beta; *R*²: determination coefficient; Δ *R*²: Changes in *R*²; GAD-7: Generalized Anxiety Disorder symptoms.

**Supplementary Table 3.** Regression models of Distress

| **Predictor** |  |  |  |  |  |  |
| --- | --- | --- | --- | --- | --- | --- |
|  | ***β*** | **B** | **T** | ***R*²** | **Δ *R*²** | ***P* value** |
|  | | |  |  |  |  |
| **Sociodemographic predictors** | | |  | .088 | .004 |  |
| Sex | -.074 | -.032 | -1.145 |  |  | .253 |
| Age | -.044 | -.001 | -.496 |  |  | .620 |
| Earn a living | .015 | -.008 | -.227 |  |  | .821 |
| Financial situation | -.056 | -.006 | -.839 |  |  | .402 |
| Professional activity(ies)  besides sports | -.068 | -.034 | -1.056 |  |  | .292 |
| Family status |  |  |  |  |  |  |
| Single | -.156 | .071 | -.710 |  |  | .478 |
| Married | .028 | .022 | .176 |  |  | .860 |
| Partnership | -.156 | -.079 | -.754 |  |  | .452 |
| Living situation |  |  |  |  |  |  |
| Alone | .086 | .043 | -671 |  |  | .503 |
| With partner | .000 | 3.432 | .000 |  |  | 1.000 |
| With partner and child(ren) | -.005 | -.005 | -.049 |  |  | .961 |
| With parents | -.060 | .029 | .467 |  |  | .641 |
| Flat sharing | .007 | .004 | .057 |  |  | .955 |
| **Sports related variables** | | |  |  |  |  |
| Years in elite sport | .081 | .003 | 1.068 |  |  | .286 |
| Number of training units  per week | .107 | .005 | 1.465 |  |  | .144 |
| Duration of training units | -.045 | .000 | -.683 |  |  | .496 |
| Type of sports |  |  |  |  |  |  |
| Ball sports | .203 | .092 | 1.450 |  |  | .148 |
| Combat sports | .170 | .142 | 1.851 |  |  | .065 |
| Strength sports | .086 | .080 | .960 |  |  | .338 |
| Track and field | .075 | .054 | .748 |  |  | .455 |
| Equestrian sports | .138 | .121 | 1.508 |  |  | .133 |
| Gymnastics | -.023 | -.025 | -.285 |  |  | .776 |
| Water sports | .185 | .086 | 1.311 |  |  | .191 |

Note. *N* = 275. *β*: standardized coefficient beta; B: unstandardized coefficient beta; *R*²: determination coefficient; Δ *R*²: Changes in *R*².

**Supplementary Table 4.** Regression models of PHQ-15

| **Predictor** |  |  |  |  |  |  |
| --- | --- | --- | --- | --- | --- | --- |
|  | ***β*** | **B** | **T** | ***R*²** | **Δ *R*²** | ***P* value** |
|  | | |  |  |  |  |
| **Sociodemographic predictors** | | |  | .296 | .231 |  |
| Sex | .248 | 2.167 | 4.352 |  |  | <.001 |
| Age | -.083 | -.057 | -1.616 |  |  | .290 |
| Earn a living | -.013 | -.145 | -.231 |  |  | .817 |
| Financial situation | -.293 | -.601 | -5.035 |  |  | <.001 |
| Professional activity(ies)  besides sports | -.072 | -.727 | -1.272 |  |  | .205 |
| Family status |  |  |  |  |  |  |
| Single | -.162 | -1.477 | -.836 |  |  | .404 |
| Married | -.072 | -1.151 | -.517 |  |  | .605 |
| Partnership | -.143 | -1.450 | -.783 |  |  | .434 |
| Living situation |  |  |  |  |  |  |
| Alone | -.065 | -.649 | -.579 |  |  | .563 |
| With partner | -.156 | -1.754 | -1.406 |  |  | .161 |
| With partner and child(ren) | -.051 | -1.076 | -.545 |  |  | .586 |
| With parents | -.223 | -2.134 | -1.957 |  |  | .052 |
| Flat sharing | .014 | .146 | .131 |  |  | .896 |
| **Sports related variables** | | |  |  |  |  |
| Years in elite sport | .059 | .048 | .885 |  |  | .377 |
| Number of training units  per week | .160 | .160 | 2.492 |  |  | .013 |
| Duration of training units | .004 | .000 | .062 |  |  | .950 |
| Type of sports |  |  |  |  |  |  |
| Ball sports | .197 | 1.795 | 1.605 |  |  | .110 |
| Combat sports | .156 | 2.619 | 1.928 |  |  | .055 |
| Strength sports | .122 | 2.292 | 1.555 |  |  | .121 |
| Track and field | .143 | 2.048 | 1.615 |  |  | .108 |
| Equestrian sports | -.097 | -1.721 | -1.210 |  |  | .228 |
| Gymnastics | .108 | 2.344 | 1.507 |  |  | .133 |
| Water sports | -.014 | -.130 | -.112 |  |  | .911 |

Note. *N* = 275. *β*: standardized coefficient beta; B: unstandardized coefficient beta; *R*²: determination coefficient; Δ *R*²: Changes in *R*²; PHQ-15: Somatic Symptom Disorder symptoms.

**Supplementary Table 5.** Regression models of SSD-12

| **Predictor** |  |  |  |  |  |  |
| --- | --- | --- | --- | --- | --- | --- |
|  | ***β*** | **B** | **T** | ***R*²** | **Δ *R*²** | ***P* value** |
|  | | |  |  |  |  |
| **Sociodemographic predictors** | | |  | .191 | .117 |  |
| Sex | .147 | 2.366 | 2.413 |  |  | .017 |
| Age | -.049 | -.061 | -.581 |  |  | .562 |
| Earn a living | .043 | -.145 | .688 |  |  | .492 |
| Financial situation | -.213 | -.601 | -3.418 |  |  | <.001 |
| Professional activity(ies)  besides sports | -.031 | -.727 | -.503 |  |  | .615 |
| Family status |  |  |  |  |  |  |
| Single | -.182 | -3.047 | -.876 |  |  | .382 |
| Married | -.130 | -3.843 | -.878 |  |  | .381 |
| Partnership | -.267 | -4.977 | -1.365 |  |  | .174 |
| Living situation |  |  |  |  |  |  |
| Alone | -.068 | -1.248 | -.566 |  |  | .572 |
| With partner | -.121 | -2.507 | -1.021 |  |  | .308 |
| With partner and child(ren) | -.099 | -3.789 | -.975 |  |  | .330 |
| With parents | -.208 | -3.672 | -1.709 |  |  | .089 |
| Flat sharing | -.103 | -1.990 | -.909 |  |  | .364 |
| **Sports related variables** | | |  |  |  |  |
| Years in elite sport | .130 | .048 | 1.826 |  |  | .069 |
| Number of training units  per week | .142 | .160 | 2.060 |  |  | .040 |
| Duration of training units | .020 | .000 | .323 |  |  | .747 |
| Type of sports |  |  |  |  |  |  |
| Ball sports | .148 | 2.473 | 1.122 |  |  | .263 |
| Combat sports | -.014 | -.439 | -.164 |  |  | .870 |
| Strength sports | .052 | 1.787 | .616 |  |  | .538 |
| Track and field | .094 | 2.483 | .994 |  |  | .321 |
| Equestrian sports | -.124 | -4.042 | -1.443 |  |  | .150 |
| Gymnastics | .106 | 4.229 | 1.380 |  |  | .169 |
| Water sports | -.013 | -.227 | -.099 |  |  | .921 |

Note. *N* = 275. *β*: standardized coefficient beta; B: unstandardized coefficient beta; *R*²: determination coefficient; Δ *R*²: Changes in *R*²; SSD12: Somatic Symptom Disorder symptoms.

**Supplementary Confirmatory Factor Analyses**

To confirm the factorial structure of the PHQ-8 in this sample of elite athletes, we performed a CFA to test the single-factor structure of the PHQ-8. The χ2-test was significant, (χ2[20] = 123.23, p < .001). The approximate fit indices used to assess model fit (RMSEA = .137, SRMR = .070, CFI = .852, TLI = .793) did not meet recommended criteria (Hu & Bentler, 1999). The single-factor structure of the PHQ-8 cannot be assumed based on the study sample. Reliability analyses revealed ω = 0.87.

CFA was used to test the single-factor structure of the PHQ-15. The χ2-test was significant, (χ2[90] = 332.47, p < .001). The approximate fit indices used to assess model fit (RMSEA = .098, SRMR = .089, CFI = .483, TLI = .397) did not meet recommended values (Hu & Bentler, 1999). The single-factor structure of the PHQ-15 cannot be assumed based on the study sample. Reliability analyses revealed ω = 0.69.

CFA was used to test the three-factorial structure of the SSD-12. The χ2-test was significant, (χ2[51] = 186.92, p < .001). The approximate fit indices used to assess model fit (RMSEA = .137, SRMR = .056, CFI = .886, TLI = .853) met recommended values (Hu & Bentler, 1999). The three-factorial structure of the SSD-12 can be assumed based on the study sample. ω was .59 for the cognitive scale, 0.87 for the affective scale and 0.83 for the behavioural scale. Overall ω was = 0.83.

CFA was used to test the single-factor structure of the GAD-7. The χ2-test was significant, (χ2[14] = 63.26, p < .001). The approximate fit indices used to assess model fit (RMSEA = .113, SRMR = .523, CFI = .930, TLI = .894) did not meet recommended values (Hu & Bentler, 1999). The single-factor structure of the GAD-7 cannot be assumed based on the study sample. Reliability analyses revealed ω = 0.88. .

CFAs could not confirm the factor structure for all instruments. However, the items of the questionnaires PHQ-15, PHQ-8 and GAD-7 were not determined in a factor-analytically guided approach but were based on the official diagnostic criteria of the assessed psychopathology.

**Supplementary Table 6. Confirmatory Factor Analyses**

| **Questionnaire** | **Model** | **Chi^2^(df)** | ***p*** | **CFI** | **TLI** | **RMSEA** | **SRMR** |
| --- | --- | --- | --- | --- | --- | --- | --- |
| **PHQ-8** | 1 | 123.23 (20) | < .001 | 0.852 | 0.793 | 0.137 | 0.070 |
| **PHQ-15** | 1 | 332.47 (90) | < .001 | 0.483 | 0.397 | 0.099 | 0.089 |
| **SSD-12** | 3 | 186.92 (51) | < .001 | 0.886 | 0.852 | 0.098 | 0.056 |
| **GAD-7** | 1 | 63.26 (14) | < .001 | 0.930 | 0.894 | 0.113 | 0.523 |

Chi²: Chi²-coefficient; *df*: degrees of freedom; CFI: comparative fit index; TLI: Tucker Lewis index; RMSEA: root mean square error of approximation; SRMR: standardized root mean square residual; PHQ-8: Depressive symptoms, GAD-7: Generalized Anxiety Disorder symptoms, SSD-12: Somatic Symptom Disorder symptoms, PHQ-15: Somatic Symptom Disorder symptoms.
